# Deep-Learning-Based Approach to Anomaly Detection Techniques for Large Acoustic Data in Machine Operation

**DOI:** 10.3390/s21165446

**Published:** 2021-08-12

**Authors:** Hyojung Ahn, Inchoon Yeo

**Affiliations:** 1 Korea Aerospace Research Institute, Daejeon 34133, Korea; 2 Fourgoodcompany Co., Ltd., Sejong 30130, Korea

**Keywords:** anomaly detection (AD), convolutional neural network, large acoustic data

## Abstract

As the workforce shrinks, the demand for automatic, labor-saving, anomaly detection technology that can perform maintenance on advanced equipment such as vehicles has been increasing. In a vehicular environment, noise in the cabin, which directly affects users, is considered an important factor in lowering the emotional satisfaction of the driver and/or passengers in the vehicles. In this study, we provide an efficient method that can collect acoustic data, measured using a large number of microphones, in order to detect abnormal operations inside the machine via deep learning in a quick and highly accurate manner. Unlike most current approaches based on Long Short-Term Memory (LSTM) or autoencoders, we propose an anomaly detection (AD) algorithm that can overcome the limitations of noisy measurement and detection system anomalies via noise signals measured inside the mechanical system. These features are utilized to train a variety of anomaly detection models for demonstration in noisy environments with five different errors in machine operation, achieving an accuracy of approximately 90% or more.

## 1. Introduction

The unexpected shutdown of a mechanical system can result in enormous costs or even serious accidents. To avoid such a case, some studies have been conducted to predict the timely and accurate diagnosis of mechanical components [1,2,3]. In fact, when diagnosing abnormalities using noise generated from a mechanical system, it is common to respond with one-shot sensory maintenance through the understanding and know-how of an experienced maintenance engineer. However, it is difficult to secure reliability for such countermeasures because objective diagnosis and maintenance cannot be performed due to the lack of certain standards or reproducibility. With recent advances in hardware and big data processing research, some approaches [4,5,6] can detect anomalies in the visual area. Taking a picture of the operation of a machine with a camera and detecting whether it malfunctions will result in deterioration of the detection quality because the operating state inside the machine cannot be extracted as image data. In many cases, visual inspection does not provide an idea of the actual condition monitored. However, cracks in certain areas inside machines, damaged fastenings of connecting parts, pumps with small leaks, friction due to unacceptable movement, etc., may not appear when inspected with a camera. To compensate for this, it is necessary to study diagnostic models [7] using signal (torque, vibration, noise, current, etc.) outputs from sensors installed in the machine to capture the machine’s operation and state. However, this method also includes uncertainty regarding the reliability of the sensor installed inside the mechanical system and has a disadvantage in that a great deal of effort and cost are required for the configuration and maintenance of the sensor in the system in order to acquire the signal. On the other hand, a method for detecting anomalies by collecting acoustic data generated in the interior space of a vehicle, such as a vehicle or aircraft directly affected by the user, may be implemented in the form of a device that is relatively easy to configure and manage. The actual condition is clarified by a clear sound pattern that is audibly monitored. Acoustic monitoring also has the advantages of involving hardware that is relatively inexpensive and easy to distribute. Early detection of malfunctioning machines with a reliable acoustic or better detection system can prevent greater damage and reduce repair and maintenance costs.

As signal processing methods and measuring instruments develop, automated inspection lines such as in automobiles and home appliances that generate noise and vibration are being explored by measuring their data using microphones or accelerometers and analyzing them [8,9]. Until now, most fault characteristics have been extracted from the data through traditional signal processing [10]. Through the signal processing result, a feature index that can analyze the characteristics of the faulty product can be calculated, and an inspection is performed through the statistical characteristics of this index. In the case of general noise, band power is also calculated through frequency analysis. When a complex failure of a bearing is included, advanced signal processing such as an envelope and a cestrum may be involved. If these automated methods are applied successfully, it is possible to perform objective and reliable tests. However, determining the inspection index in the process of automating the noise and vibration quality inspection is not easy and requires a lot of time and effort. For this reason, in recent years, deep learning models have been used to minimize the complexity of data preprocessing, feature extraction, and feature selection [11,12].

In this paper, we provide an efficient method that can learn acoustic data measured by using many microphones for detecting abnormal operations inside a machine during operation in deep learning quickly and with high accuracy. In previous studies [8,13], a single microphone or a unidirectional microphone array have been used to measure noise in a vehicle and power tools. However, because a person riding a vehicle experiences noise from all directions in a complex way, to reflect this realistically, a microphone with a spherical structure was installed to acquire acoustic data on noise from all directions in this study. Noise generated from a total of eight directions was acquired as acoustic data, similar to the operating environment of a vehicle in motion; therefore, it was possible to prevent an increase in verification costs due to the attachment of additional sensors or an AD method through sensors. Detecting AD through noise from eight directions can dramatically reduce the cost of AD verification and is the best way to increase the emotional satisfaction of users.

We propose an AD algorithm that can overcome the limitations of noise in measurements and detect system anomalies through noise signals measured inside a mechanical system. To improve the diagnostic performance, we present an anomaly diagnosis and identification model that combines the noise training data processed in various forms, a diagnosis unit applying convolutional neural net (CNN)-based mixed models, and a fault identification unit using the diagnosis results.

## 2. Related Work

For AD using deep learning, two approaches are mainly used: supervised learning and unsupervised learning. To determine normal and abnormal classifications, the data labeled in each state must be prepared in a balanced manner. Some studies [14,15,16] assumed a situation in which data for all states are sufficiently secured and solved the problem of detecting (classifying) each state from the viewpoint of classification by adopting a support vector machine (SVM) as a learning model. Glowacz et al. [13] classified acoustic feature vectors with the nearest neighbor classifier and naïve Bayes classifier. Another prior study [11] presented a method for labeling test data obtained through multiple flight tests as normal and abnormal using the K-means method to generate balanced learning datasets and then used that dataset to classify states in real time based on the linear regression method. However, in a real environment, the anomaly situation does not appear more often than the normal situation; thus, it is difficult to collect sufficient data on the anomaly state. Therefore, the learning model cannot be given a sufficient opportunity to learn the characteristics appearing in the anomaly state. In addition, it is difficult to predict what features can be extracted and how well the available features can be extracted. In this case, the problem is solved mainly from the viewpoint of AD, not from the viewpoint of classification.

Some studies [12,17,18] have classified abnormal states with the concept of reconstruction errors that were distinguished from normal states. The detection method using reconstruction error can be used for AD using accumulated test data or the real-time result, although it is weaker than the method using prediction error to predict the diagnosis result. The AD method using normal data is based on a prediction error and calculated as the difference between observed data and prediction results learned with normal data [19,20,21]. For this, LSTM [22] models based on recurrent neural networks have been widely used. This method has been applied in various studies in various forms according to the purpose in conjunction with several other learning models.

Shi et al. [23] proposed a convolutional LSTM (ConvLSTM) framework. This replaces the matrix multiplication with convolutional operation in LSTM, and the most representative form is the last layer of the CNN being composed of LSTM. This configuration has the advantage of being able to consider the temporal characteristics of features extracted by CNNs. Kim et al. [24] extracted spatial features from time series data by using a CNN in a previous step. They performed AD in web traffic by passing these features through the LSTM at the last layer. Le et al. [25] classified soluble N-ethylmaleimide-sensitive factor activating protein receptors (SNAREs) and non-SNAREs with CNNs (to train deep learning model from the generated vectors) using extracted features by the fastText model [26] (to train the vector model). To enhance prediction accuracy for time series data in multiple states, Maya et al. proposed delayed LSTM (dLSTM) based on prediction errors [20]. dLSTM is composed of two main features (Single and Predet) to verify the effectiveness of multiple models and delayed prediction [20]. They provided multiple predicted candidates by delaying the timing and selecting the values closest to the measured value as the predicted values.

To display the spatial information of image data and apply a sequential module to model the temporal correlation for a series of frames, a structurally combined framework between LSTM and the generative model is also used. This allows unsupervised diagnostics to be performed in cases where there are no labeled data for training. Lin et al. [27] used a variational autoencoders (VAE) module for forming robust local features over short windows and an LSTM module for estimating the long-term correlations in the series on top of the features inferred from the VAE module. P.T. et al. [28] estimated and analyzed the accuracy of each model by adopting different deep learning approaches, such as feed-forward neural networks (FFNN) and recurrent neural network (RNN), using data obtained from on board vessels, and comparing them with actual measurements. The work of P.T. et al. therefore suggested how researchers could determine an efficient deep learning model for heterogeneous data sources. Le et al. [29] compared the performances for five different predictive modeling (KNN, random forest (RF), SVM, multi-layer perceptron (MLP), CNN) to show that deep neural networks could be a better choice for DNA sequencing dataset because of a sufficient amount of training data and the possibility of generating hidden features of deep neural networks [29].

This study seeks to compare performance by adopting representative methodologies between two major AD approaches supervised learning and unsupervised learning to predict diagnostic results by processing large capacity noise data. We also want to discuss how deep learning, a state-of-the-art technology, reduces the space on a dataset while maintaining the characteristics of a time series sound dataset, as it lacks the memory capacity required to directly train large amounts of acoustic data collected over multiple channels.

## 3. Materials and Methods

### 3.1. Acoustic Data Measurements for Anomaly Detection

The measurement system was designed and configured to be easily applied in mass production lines. In the case of mass production lines, because production facilities are operated 24 h a day, 365 days a year, durability and performance are very important in measurement and analysis systems. Therefore, the measurement system was selected and configured as a product that did not cause errors in measurement even after years of use. A microphone is basically used for data measurements; therefore, a model capable of sampling up to 102,400 Hz per second was selected so that the measurable frequency could be analyzed at a maximum of 20,000 Hz or higher. The input of the product for inspection and the start of measurement were provided by the PLC as a digital signal; therefore, a digital input module was configured and selected, as detailed in Table 1.

**Figure 1:**
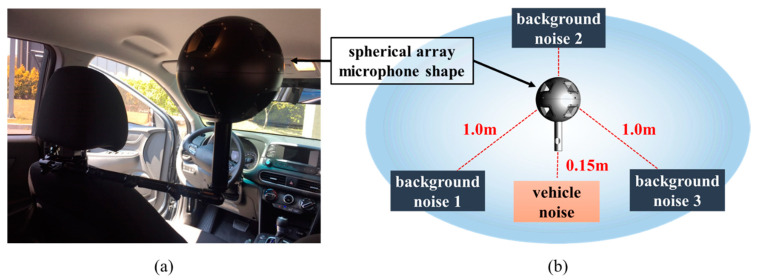
Experimental set-up: (**a**) measurement environment, (**b**) data acquisition background noise locations and environment configuration.

When noise came from various directions, the system was designed to improve the directionality of the microphone so that the location of the noise could easily be identified. Finally, a spherical array that could capture the most noise was fabricated. The spherical array microphone was designed to be applicable according to the measurement conditions by creating two types, 170 and 240 mm in diameter, as shown in Figure 1.

A spherical array microphone, such as that shown in Figure 1a, was installed inside a vehicle to measure the noise transmitted to inside the cabin while the vehicle was running. Data were obtained in two ways. The first was to acquire data through a multi-channel microphone by outputting only product noise through a speaker without background noise. Secondly, the product noise and three background noises were simultaneously output to acquire data through a multi-channel microphone. Therefore, the noise data were stored as sound data, with a sampling size of 51.2 kHz. In the case of background noise samples, #1 (dialog sound), #2 (music), and #3 (factory noise) were measured to create an environment through repeated playback. An environment in which such a noise may be generated is similar to the sound generated while an actual vehicle is being driven, and an environment is created to detect the abnormal operation of the vehicle from the noise generated in such an environment, as shown in Figure 1b.

### 3.2. Dataset

In this study, to build a learning model that can predict and identify failures, the signal strength (%) was changed for each driving condition (road surface condition, driving speed, etc.) and the failure data were set for each failure part. Additionally, the acoustic data consisted of recordings of normal or abnormal behaviors of five vehicle components (step bar links, tailgate guides, lower arms, shock absorbers, and tie rods). There were five datasets that showed different anomalous behaviors for each type of action. In addition, background noise recorded while operating the actual machine was added to each recording based on a specific SNR (signal-to-noise ratio). The analysis used a sound with an SNR of 6 dB. This is remarkably close to actual use because the microphone monitoring machine cannot prevent it from capturing background noise in the machine’s operating environment. Each channel length (*N_t_*) was 51,200.

The data file generated by receiving the operation sound of the machine via 8 channels (*N_s_*) consisted of 5 error operations that were judged to be abnormal, and one operation of the machine was confirmed normally. The classifications of data are shown in Table 2.

**Figure 2:**
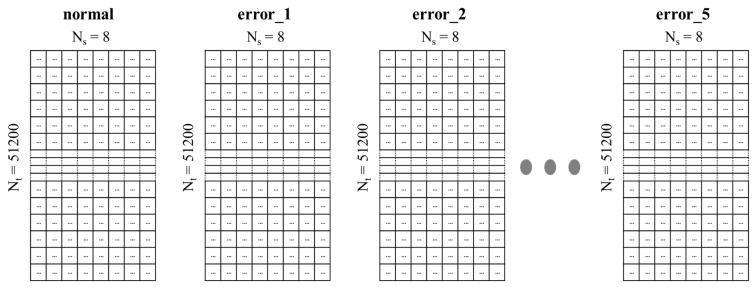
Structure of an acoustic dataset with a size of (51,200 × 8).

In this study, the motion sound of the machine was collected using a spherical microphone with 8 acoustic inputs, and about 13,000 data points were collected, as shown in Table 2. Therefore, a data file consisted of 8 channels, the size of a file was about 3.5 MB, and it had a structure of (51,200 × 8), as shown in Figure 2.

However, because each data file was 3.5 MB, the total amount of data to be trained look up 13,162 × 3.5 MB about 50 GB of space. Training 50 GB of data requires considerable computing resources (computational power and memory capacity), and the training period would take from a week to month. In this study, we propose a method of extracting features of large-capacity data for large-capacity anomaly detection, converting these features into 2D images, and then performing learning.

### 3.3. Feature Extraction

**Figure 3:**
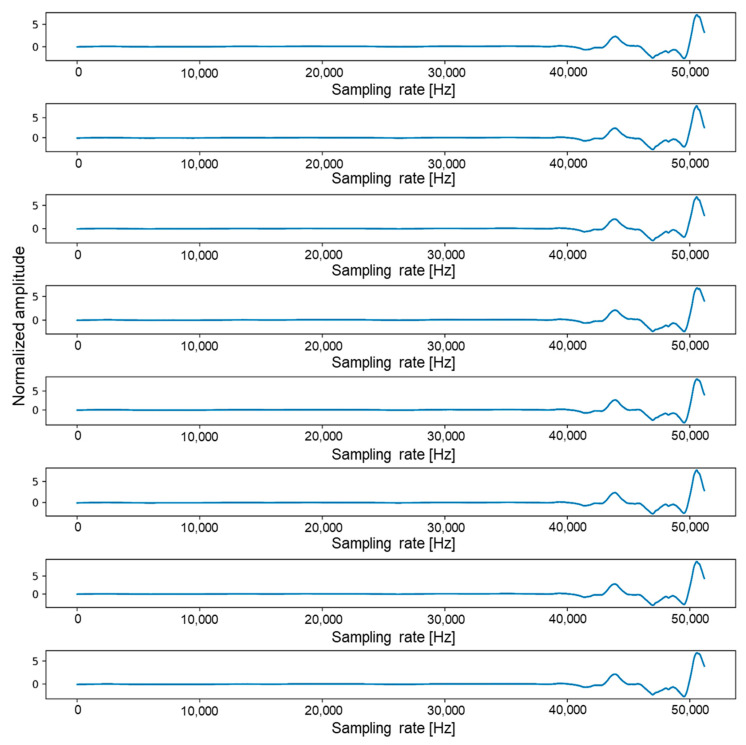
Graph of one dataset with sample size of 51,200 and 8 channels.

To use all the data in Table 1 for training, we need a large amount of data and memory space, although too much training data will significantly slow down the overall training rate. In this study, we converted one data file equivalent to 3.12 M per file into a 0.00772 MB image file with 8 sound channels. By reducing the size of each file by about 400 times, the training speed was increased by tens of times. An 8-channel sound pattern, which took up about 3.12 MB of space per file, could be plotted using Python’s plot function, as shown in Figure 3. If we transpose acoustic data composed of 51,200 × 8 to 8 × 51,200, it is as shown in Figure 3. Figure 3 consists of a total of 8 plots, and each plot represents one channel. In each plot, the x-axis has a range from 0 to 51,200, and the y-axis has a range from −5 to 8.

**Figure 4:**
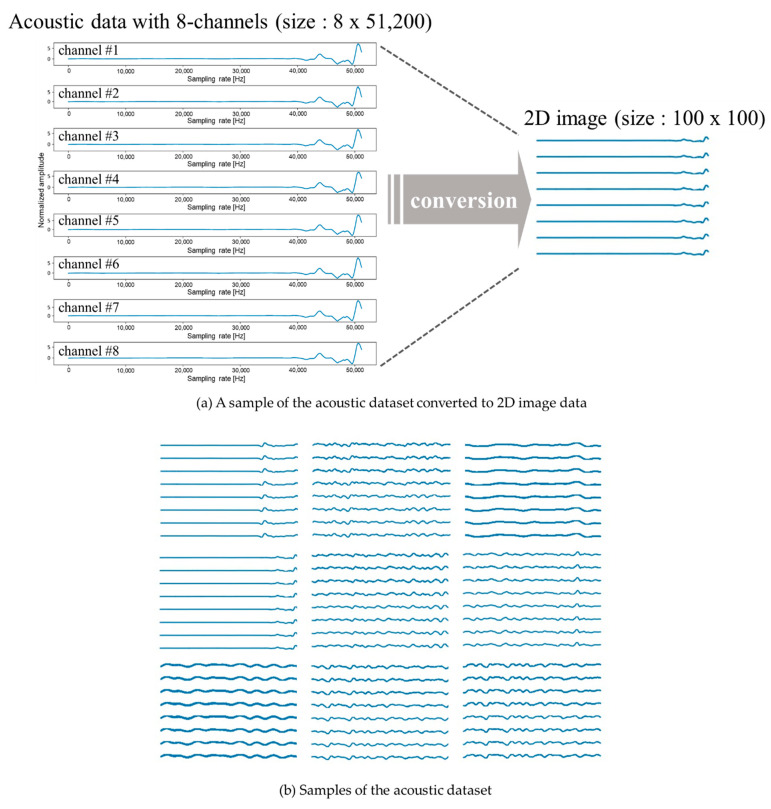
A sample of the acoustic dataset converted to 2D image data.

When features were extracted from the (51,200 × 8) dataset graph and converted into a 2D image, the acoustic data were converted into a 2D image, as shown in Figure 4. To increase the speed, we reduced the size of the 2D image to (100 × 100), thus converting a total of 13,000 sound data values into 13,000 2D images of size (100 × 100).

## 4. Anomaly Detection Deep Learning Models

**Figure 5:**
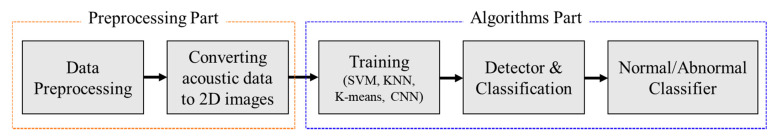
Block diagram of AD models.

This study compares four well-established AD algorithms. To accurately classify abnormal data from a large-scale acoustic dataset, a supervised classification approach and an unsupervised clustering approach were applied. SVM [30], K-means [31], and KNN [32], which are representative algorithms for classification and clustering, were compared with the CNN to confirm the accuracy of AD and the classification of large-scale accounting data. CNNs are known to perform well on two-dimensional data inputs with spatial relationships [33]. As shown in Figure 5, the first task is to transpose and normalize 13,000 acoustic data to train and test. It reads the preprocessed data one by one and converts them into a 2D image format. After this conversion is completed, the preparation process for training is completed. Next, according to the four AD algorithms, one normal and five abnormal operations are classified.

This study applied an architecture that integrated CNN and gated recurrent unit (GRU) [34] deep learning models. When working with a typical neural network, the input data have to be transformed into a single vector that acts as an input to the neural network and traverses the layers of the neural network. Each neuron in each layer is interconnected with all neurons in the previous layer. In other words, the neurons within each layer are not connected to each other. They are only connected to neurons in adjacent layers. The output layer, the last layer in the network, represents the final output. Within the deep CNN model used, a layer takes inputs from a set of units located in smaller neighbors of the previous layer. Using the local receptive field, neurons can extract the basic function of the input and then combine it with the function of the upper layer. The outputs of these sets of neurons constitute the functional map. For each functional map, we implemented this procedure sequentially, scanning the input data into a single neuron with a local receptive field and storing the state of this neuron at its location in the functional map. Limited units of functional maps do the same in different instances, and multiple functional maps (including weight vectors) can form one convolutional layer. Therefore, we could extract multiple features from each instance [35]. The model was comprised of six main layers: an input layer, three two-dimensional convolutional layers, a fully connected layer, and an output layer. We applied a dropout [36] layer between the convolutional layers and optimized the system parameters, as shown in Table 3, for parameter details. Optimal system parameters were identified using a trial-and-error method over multiple different parameter ranges consisting of kernel size, number of filters, learning rate, batch size, and training epochs for each normal and abnormal category and then evaluated their performance based on a classification accuracy. We used 80% of the randomly selected data samples for training the model, and the remaining 20% for the validation process; the ratio was the result of a trial/error experience within the model configuration tuning. After each epoch (an epoch is one complete presentation of the sample dataset to train a machine learning model), we monitored the performance of the model to make sure that the model stopped training at the minimum validation loss to avoid the possibility of overfitting.

**Figure 6:**
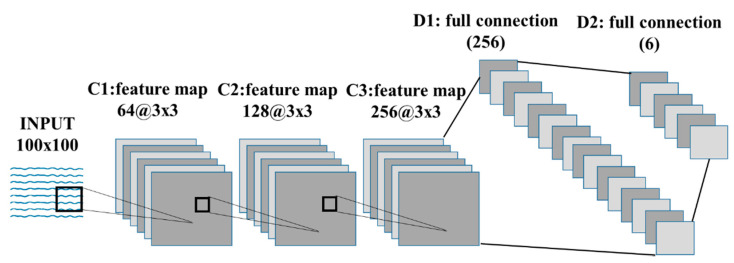
Deep learning architecture for anomaly detection.

To build a deep CNN, we applied convolutions to the input function and kernel elements as shown in Figure 6.

In this study, the kernel size (convolution window) used was 3 × 3 for the 3 2D convolution layers, and the result of the convolution operation was sent to the activation function.

The last feature was the activation function (rectified linear unit, ReLU) [37] applied to the convolutional output (i.e., 3D tensor). We connected the output portion of the CNN layer to the deep neural network (DNN) [38] input to classify one normal and five abnormal states. Traditional neural networks such as CNNs assume that all inputs and outputs are independent. Therefore, when the previous information is essential, the DNN architecture combined with the CNN architecture makes the previous information available as an input, generating more accurate prediction values. Finally, softmax [39] is applied to extract the feature vectors.

In this study, we used the F1 score method to compare the accuracy of the tests for the four algorithms. The F1 score is the F-score or F-measure in statistical analyses of binary classifications, and is a measure of the accuracy of a test [40]. This is calculated from the precision and recall of the test, where precision is the number of true positive results divided by the number of all positive results, including those that were not accurately identified, and recall is the number of true positive results divided by the number of all samples that must be confirmed as positive. Precision is also referred to as the positive predictive value and recall is also referred to as the sensitivity of diagnostic classification.

The accuracy is the number of correctly predicted data divided by the total amount of data:

(1) $$\mathrm{accuracy}=\frac{\mathrm{TruePositives}+\mathrm{TrueNegatives}}{\mathrm{TruePositives}+\mathrm{TrueNegatives}+\mathrm{FalsePositives}+\mathrm{FalseNegatives}}$$

**Figure 7:**
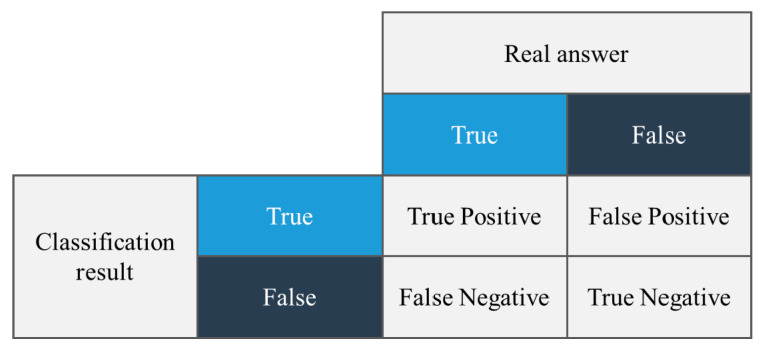
The terminology used to calculate the accuracy.

The terminology used in the formula for accuracy can be easily understood by examining Figure 7.

Recall is the number of actual true data that the model recognizes as true data, as shown in Equation (2).

(2) $$\mathrm{Recall}=\frac{\mathrm{TruePositives}}{\mathrm{TruePositives}+\mathrm{FalseNegatives}}$$

Precision is the number of actual true data that the model predicted to be true:

(3) $$\mathrm{Precision}=\frac{\mathrm{TruePositives}}{\mathrm{TruePositives}+\mathrm{FalsePositives}}$$

Precision and recall have a trade-off relationship and are used to measure model performance. However, we still require one more indicator to explain how effective the model is. In this case, the F1 score is used. As shown in Equation (4), the F1 score is the harmonic mean of precision and recall. The highest possible value of the F1 score is 1.0, which represents perfect precision and recall, and 0 is the lowest possible value if precision or recall are zero:

(4) $$F_{1}=\frac{2}{\mathrm{recall}{}^{-1}+\mathrm{precision}{}^{-1}}=2\cdot \frac{\mathrm{precision}\cdot \mathrm{recall}}{\mathrm{precision}+\mathrm{recall}}$$

## 5. Results and Discussions

In this study, six groupings (one normal and five abnormal) were performed on 200, 500, 1000 and 10,000 datasets. When Support Vector Machine (SVM) is given a dataset that falls into one of the two categories, the SVM algorithm uses the specified dataset to add new data to any category. It creates a non-probabilistic binary linear classification model that decides whether to do so or not. The generated classification model is represented by the boundary of the space to which the data are mapped, and the SVM algorithm is an algorithm that finds the boundary of the largest width among them. SVM can be used for linear as well as non-linear classification. K-means clustering aims to split n observations into k clusters. Each observation belongs to the cluster with the closest mean (cluster center or cluster center). K-means clustering minimizes the intra-cluster variance (square Euclidean distance), but not the normal Euclidean distance. Therefore, the mean optimizes the squared error, whereas only the geometric median minimizes the Euclidean distance. KNN (K-Nearest Neighbor) is a type of algorithmic awareness learning that uses labeled data to perform classification tasks. As the name of the algorithm suggests, it is an algorithm that performs classifications by referring to the labels of *k* different data that have a close distance from the data. Distances are mainly measured using the Euclidean distance calculation method, but the larger the vector size, the more complex the calculation. KNN looks at the labels of surrounding *k* points and predicts the input with the most labels. A CNN using an Adam optimizer [41] trained batch sizes of 50 and 150 epochs.

The dataset used was divided into five groups according to the amount of data, and the ratio of training data and test data in each group was set to 4:1; the amount of data used is shown in Table 4. The datasets used for training and testing were divided into five groups, ranging from 200 to 10,000, and the data that could not be converted into 2D images among the total data of each dataset were excluded from the test dataset.

### 5.1. Results of the SVM

Table 5 shows the measurement statistics of the results for the SVM and the accuracy of AD using datasets of various sizes.

**Figure 8:**
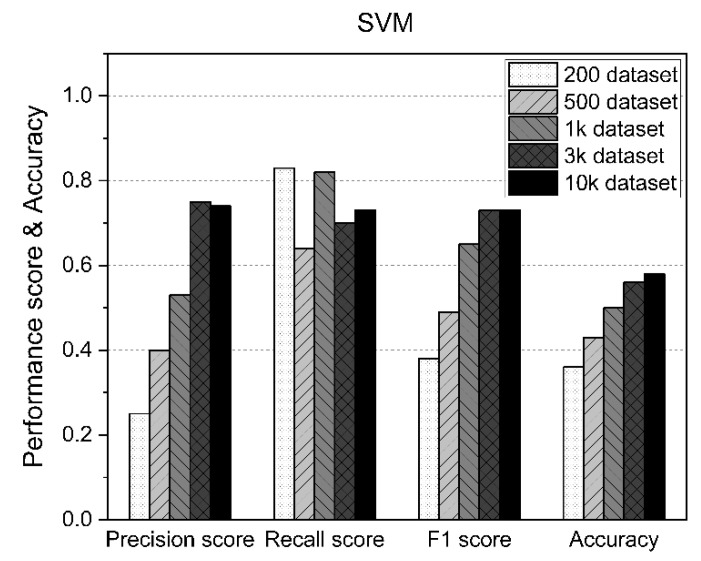
SVM performance results according to the size of the dataset.

In the SVM approach, the F1 score and accuracy increased as the size of the dataset increased, although as shown in Figure 8, the accuracy exceeded 50% from the results for 1000 datasets, but also about 58%, even when using up to 10,000 datasets.

### 5.2. Results of K-Means

**Figure 9:**
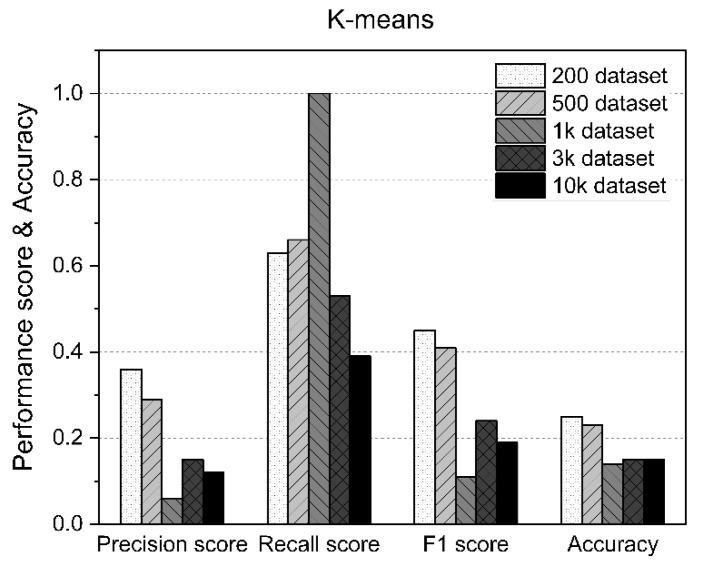
K-means performance results according to the size of the dataset.

The main limitation of K-means is that it is a cluster model. This concept is based on separable spherical clusters, where the mean converges around the cluster center. The clusters are expected to be of similar sizes; therefore, assignment to the nearest cluster centroid is the correct assignment. In this study, when applied to a dataset with K = 3 values, the results often failed to separate the three errors contained in the dataset. If K = 2, two visible clusters were found, whereas if K = 3, one of the two clusters was split into two even parts. In fact, K = 2 was a better fit for this dataset, even though it contained three classes. Similar to other clustering algorithms, the K-means results assume that the data meet certain criteria. It works well on some datasets but fails on others. Table 6 shows the measurement statistics of the results for K-means and the accuracy of AD using datasets of various sizes. Figure 9 shows the worst K-means results of the methods used in this study.

### 5.3. Results of the KNN

**Figure 10:**
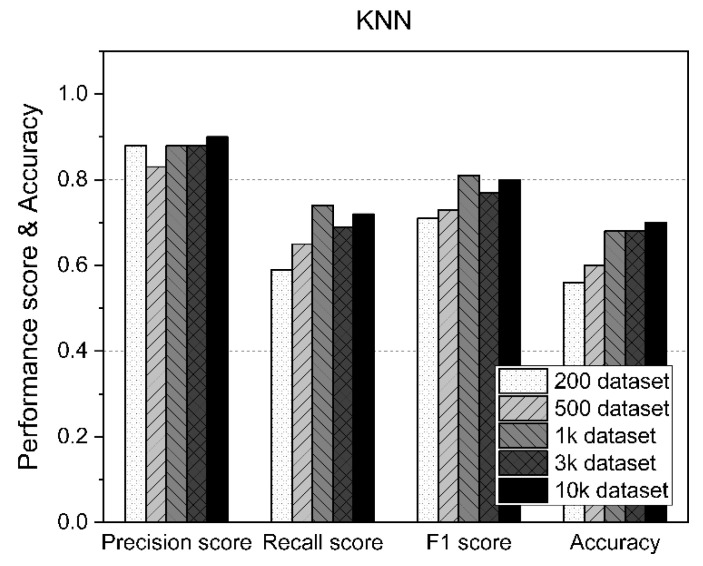
KNN performance results according to the size of the dataset.

Table 7 shows the measurement statistics of the results for KNN and the accuracy of AD using datasets of various sizes. As shown in Figure 10, KNN generally performed consistently regardless of the size of the dataset. However, despite using 10,000 datasets, the accuracy was only about 70%.

### 5.4. Results of the CNN

**Figure 11:**
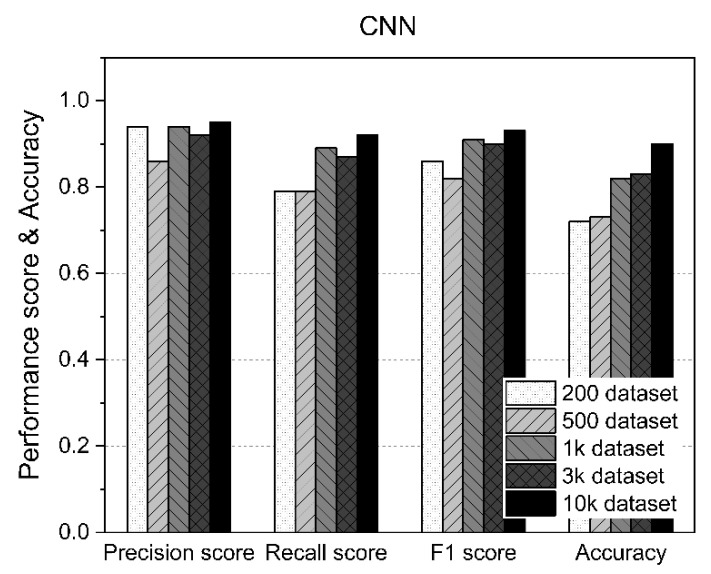
CNN performance results according to the size of the dataset.

Table 8 shows the measurement statistics of the results for CNN and the accuracy of AD using datasets of various sizes. As shown in Figure 10, the CNN improved the accuracy of AD as the number of datasets increased. Using fewer than 1000 datasets, an accuracy of about 70% was achieved, whereas an accuracy of 80% or higher was achieved for datasets of 1000 or more. In particular, as shown in Figure 11, the case with 10,000 datasets showed 90% accuracy. Therefore, when the CNN was used, the F1 score and accuracy improved because the amount of data used for training increased.

### 5.5. Comparison of F1 Score and Accuracy for Four Models

The advantage of SVMs is that they are less prone to overfitting and are easier to use than neural networks. However, in order to find the optimal model, various tests for the kernel and the model are required. Additionally, when there are many input datasets, the learning speed is slow. K-means is a relatively simple algorithm and can find meaningful structures without prior information about a given dataset. However, different results may appear depending on the initialization, and in the worst case, it may fall into the local optima. KNNs are simple, efficient, and fast to train. However, since it does not create a model, it is limited in understanding the relationship between features and classes. In addition, rather than identifying important variables through KNN, people have to select variables that they think are important, so a lot of preliminary analysis is required. CNN is one of the most popular deep learning algorithms in the image processing field. The concept of deep learning algorithm has the disadvantage that it cannot analyze the inside because the internal structure is processed as a black box, but it shows excellent results in the function of extracting and classifying or comparing the features of the learned image data. The disadvantage of CNN is that it is not easy to accurately set various hyperparameters, and the results may vary depending on the hyperparameters.

**Figure 12:**
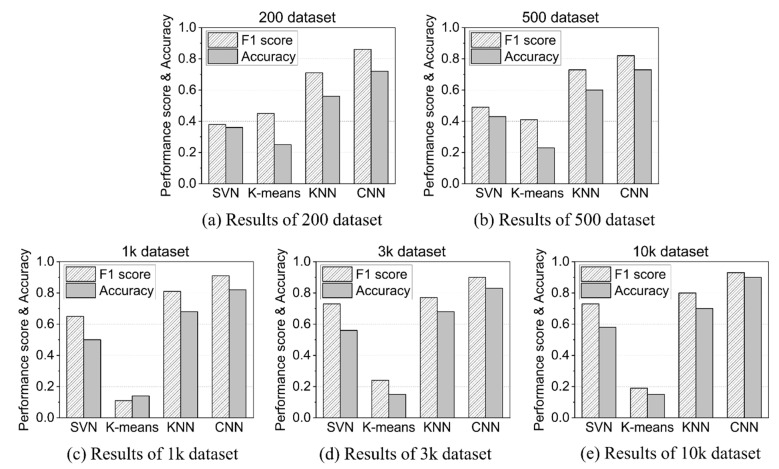
F1 score and accuracy comparison for four models.

As shown in Figure 12, the CNN performance was overwhelmingly better than the other three models, regardless of the size of the dataset. The reason for this is that CNN’s 2D image learning performance is excellent for 2D images extracted from the characteristics of time series acoustic data. In the case of CNN, the larger the dataset size, the more its performance was improved, and when using 10,000 datasets, which was the maximum dataset size, the accuracy of AD reached 90%.

### 5.6. Discussions

For the AD algorithm of acoustic data, we initially selected a combination of 1D-CNN and LSTM. However, it was impossible with our experimental equipment to train more than 13,000 datasets and total acoustic data of about 50 GB with the combination of 1D-CNN and LSTM. A method for learning by transforming a 1D dataset, which is often used in the research field of computer image processing [42], was able to reduce the storage capacity to 1.7 GB. If the dataset capacity is reduced, there is a possibility that small changes in the data can be removed. However, because acoustic data were used in this research, only the characteristics of the frequency pattern of the acoustic data could be trained as features. Therefore, the training was possible with little loss of frequency characteristics of acoustic data.

The disadvantage of this study is that the method of detecting normal data and abnormal data in this study consists of one normal label and a method of classifying five abnormalities. Although the 13,000 datasets used in this study constitute a lot of data, the method of detecting errors with already acquired normal and abnormal datasets makes it difficult to apply them in real time. Ultimately, to become an AD method that can be applied when a machine operates in real time, it should be a method of classifying only normal and abnormal operations for acoustic data input in real time and classifying the type of abnormality only when it is abnormal. To apply this improved method, more acoustic datasets are needed, and using this data, a more efficient learning environment (more training examples, more input variables, larger input size, etc.) can be used to efficiently train deep learning algorithms [43]. Another disadvantage of this approach is that there is no standard procedure for determining the optimal network architecture (e.g., number of hidden layers/units, training parameters). This is usually determined by trial and error and can have a significant impact on the performance of the model. These hyperparameters have internal dependencies and are therefore very expensive to tune [44]. Another drawback of this study is that after converting the acoustic data into 2D images, the CNN model was trained using the image data. Therefore, unlike the model that uses acoustic data directly, the CNN model cannot consider the inherent characteristics of acoustic data unless an appropriate index is used within the input. For example, sound interference between eight channels or unique characteristics of a specific frequency region cannot be considered. Deep learning processes extract essential features from raw input data through a greedy layer-wise learning process [45]. Thus, the algorithm develops a layered architecture to represent the data and shows the impact of each feature [46]. However, deep learning models such as CNNs can only be trained on historical data for the feature extraction process. Therefore, the sensitivity of the input parameters of the CNN model to the output is likely to be set disproportionately due to the learning of past data. Anomalies in these trends can lead to disproportionate prediction sensitivities at different levels of input parameters. The technical part to be improved in this study is that it is closer to anomaly classification rather than anomaly detection because it does not classify normal and abnormal datasets but classifies one normal dataset and five abnormal datasets.

Therefore, future improved research will focus on anomaly detection, which detects normal and abnormal operations, and plan to classify abnormalities only in the case of abnormalities. In this way, more accurate anomaly detection is possible by separating anomaly detection and anomaly classification.

## 6. Conclusions

In this study, in order to detect abnormal operation of the machine, an omnidirectional (eight-channel) microphone was installed to reduce operating noise of the machine in the same way as in the environment perceived by the user, instead of the existing complicated method of installing various sensors or measuring instruments. We have proposed a user-friendly AD method that learns the operating noise generated in such an environment, detects anomalies in machine operation, and distinguishes abnormalities caused by moving parts. While the machine is running, eight operating sounds were captured to detect the abnormal operation of five main components, forming an eight-channel dataset. However, this captured dataset occupied about 50 GB of capacity; thus, training with a typical deep learning algorithm would require too much time and memory space. In this study, we preprocessed a time series sound dataset into a 2D-based image dataset to speed up the training of a deep learning neural network on a sound-based machine motion AD dataset and reduce the memory footprint required. While maintaining the characteristics of the time series sound dataset, the space of the dataset was reduced to 1.67 GB, resulting in a training time that is about 10 times faster. We explored various deep learning algorithms to train a preprocessed machine AD 2D dataset. Deep convolutional neural networks split the images into smaller kernels for training, thus transforming eight-channel time series sounds into 2D images, to capture changes in a machine’s operational acoustic dataset more accurately. We used a deep convolutional neural network to detect anomalies in our machines, and when training 10,000 out of about 13,000 complete datasets, the accuracy of the AD was close to around 90%. However, the method used in this study classified a normal dataset and five abnormal datasets by training all of them. The weakness of this approach is that it cannot be properly classified when additional abnormal data are generated alongside the five abnormal datasets. To overcome this limitation, we plan to improve this study to detect only normal and abnormal data and to classify what type of abnormal data are present if these data are classified as not being normal. In the future, to extend beyond the detection and classification of the above operations to the prediction and identification of abnormal operations, improvements will be made to apply the GAN-LSTM method based on the time characteristics of the eight-channel time series sound datasets.

## Figures and Tables

**Table 1:** Measurement system configuration and detailed specifications.

Name	Model	Manufacturer	Specifications
Chassis	NI cDAQ-9189	NI	8-Slot, Extended Temperature Ethernet Compact DAQ Chassis
Noise measurement module	NI 9232	NI	Number of input channels: 3 ch Sampling Rate: 102.4 kS/s/ch Input Voltage: ±30 V C Series Sound and Vibration Input Module
Digital input module	NI 9421	NI	Input Voltage: 24 V Number of input channels: 8 ch (sinking input) Measurement speed: 100 μs C series digital module
Microphone	130F22	PCB	Diameter: 1/4 inch Sensitivity: 45 mV/Pa Frequency Response (±4 dB): 10 to 20,000 Hz

**Table 2:** Number of datasets for anomaly detection.

# of Files	Normal	Error #1	Error #2	Error #3	Error #4	Error #5
13,162	6104	3760	394	903	1274	727

**Table 3:** Parameters of the AD algorithms.

Classifier	Parameters
SVM	random_state = 42, max_iter = 100,000
K-means	n_components = 300
KNN	k = 6
CNN	n_filters = 16/64/64, dropout = 0.25, optimizer = adam

**Table 4:** The number of training data and testing data in the dataset used in the experiments.

	# of Training Data	# of Testing Data	# of Total Data
200 Dataset	160	36	196
500 Dataset	400	99	499
1k Dataset	800	196	996
3k Dataset	2400	583	2983
10k Dataset	8000	1236	9236

**Table 5:** Results of the SVM.

	Precision Score	Recall Score	F1 Score	Accuracy
200 Dataset	0.25	0.83	0.38	0.36
500 Dataset	0.4	0.64	0.49	0.43
1k Dataset	0.53	0.82	0.65	0.5
3k Dataset	0.75	0.7	0.73	0.56
10k Dataset	0.74	0.73	0.73	0.58

**Table 6:** Results of K-means.

	Precision Score	Recall Score	F1 Score	Accuracy
200 Dataset	0.36	0.63	0.45	0.25
500 Dataset	0.29	0.66	0.41	0.23
1k Dataset	0.06	1	0.11	0.14
3k Dataset	0.15	0.53	0.24	0.15
10k Dataset	0.12	0.39	0.19	0.15

**Table 7:** Results of the KNN.

	Precision Score	Recall Score	F1 Score	Accuracy
200 Dataset	0.88	0.59	0.71	0.56
500 Dataset	0.83	0.65	0.73	0.6
1k Dataset	0.88	0.74	0.81	0.68
3k Dataset	0.88	0.69	0.77	0.68
10k Dataset	0.9	0.72	0.8	0.7

**Table 8:** Results of the CNN.

	Precision Score	Recall Score	F1 Score	Accuracy
200 Dataset	0.94	0.79	0.86	0.72
500 Dataset	0.86	0.79	0.82	0.73
1k Dataset	0.94	0.89	0.91	0.82
3k Dataset	0.85	0.88	0.86	0.83
10k Dataset	0.95	0.92	0.93	0.90

## Data Availability

The source codes used to support the findings of this study are available from the first author upon request.
